# Inflammatory demyelination induces glia alterations and ganglion cell loss in the retina of an experimental autoimmune encephalomyelitis model

**DOI:** 10.1186/1742-2094-10-120

**Published:** 2013-10-04

**Authors:** Lioba Horstmann, Heiko Schmid, André P Heinen, Florian C Kurschus, H Burkhard Dick, Stephanie C Joachim

**Affiliations:** 1 Experimental Eye Research Institute, Ruhr University Eye Hospital, In der Schornau 23-25, 44892 Bochum, Germany; 2 Institute for Molecular Medicine, University Medical Center of the Johannes Gutenberg University, Obere Zahlbacherstrasse. 67, 55131 Mainz, Germany

**Keywords:** EAE, MOG, Multiple sclerosis, Optic neuritis, Glia, Demyelination, Optic nerve, Retina, RGC, Apoptosis

## Abstract

**Background:**

Multiple sclerosis (MS) is often accompanied by optic nerve inflammation. And some patients experience permanent vision loss. We examined if the grade of optic nerve infiltration and demyelination affects the severity of clinical signs in an experimental autoimmune encephalomyelitis (EAE) model. The loss of retinal ganglion cells (RGC) and alterations in glia activity were also investigated.

**Methods:**

C57BL/6 mice were immunized with peptide MOG_35-55_ in complete Freund’s adjuvant (CFA) and controls received PBS in CFA. Then 23 days post immunization eyes were prepared for flatmounts and stained with Nissl to evaluated neuronal density. Clinical EAE symptoms as well as cell infiltration and demyelination in the optic nerve were examined. Retinal sections were stained with hematoxylin and eosin and silver stain. Immunohistochemistry was used to label RGCs (Brn-3a), apoptotic cells (caspase 3), macroglia (glial fibrillary acidic protein (GFAP)), microglia (Iba1), macrophages (F 4/80) and interleukin-6 (IL-6) secretion.

**Results:**

EAE symptoms started at day 8 and peaked at day 15. Cell infiltrations (*P* = 0.0047) and demyelination (*P* = 0.0018) of EAE nerves correlated with the clinical score (*r* > 0.8). EAE led to a significant loss of RGCs (*P*< 0.0001). Significantly more caspase 3^+^ cells were noted in these animals (*P* = 0.0222). They showed an increased expression of GFAP (*P*< 0.0002) and a higher number of microglial cells (*P*< 0.0001). Also more macrophages and IL-6 secretion were observed in EAE mice.

**Conclusions:**

MOG immunization leads to optic neuritis and RGC loss. EAE severity is related to the severity of optic nerve inflammation and demyelination. EAE not only affects activation of apoptotic signals, but also causes a glial response in the retina.

## Background

Multiple sclerosis (MS) is an autoimmune-mediated neurodegenerative disease with characteristic inflammatory demyelination in the central nervous system [1]. Patients with this disease suffer from many disabilities like memory dysfunction, cognitive deficit and movement disorders. Studies on patients indicate significant axonal damage and a decrease of retinal ganglion cells accompanied with a loss of vision [2-4] and thinning of the retinal nerve fiber layer. Decreased macular volume can be seen after a single clinical episode of optic neuritis, which is correlated with the level of residual visual dysfunction [5]. The majority of patients recover from this vision loss after several weeks. But in 40% of these patients vision loss remains permanent [6,7]. Studies indicate that on the one hand the loss of vision after an episode of optic neuritis correlates with death of retinal ganglion cells (RGCs) [8,9], on the other hand there are some patients where RGC loss occurs without an episode of optic neuritis, suggesting that the RGC decrease mediates visual loss in this disease [10]. The precise mechanisms leading to this neurodegeneration remain unknown.

The experimental autoimmune encephalomyelitis (EAE) model is the most common animal model used for multiple sclerosis studies [3,6,11,12]. We immunized C57BL/6 mice with myelin oligodendrocyte glycoprotein peptide (MOG_35–55_), a widely applied method to induce EAE in animals [1,5,12,13]. Both MS and EAE are characterized by inflammation and neurodegeneration in the spinal cord and the brain [12]. Although, there are some studies describing the effect of MS and EAE in the optic nerve, the impact on the retina is still not well understood [14-16].

In our study we examined optic nerve cell infiltration to determine if the severity of the optic nerve histopathology correlates with the clinical EAE score. Additionally, eyes were evaluated for RGC loss and alterations in micro- and macroglial activation. Furthermore, we checked for activated phagocytic microglial cells and interleukin-6 (IL-6) as signs of inflammation. The severity of cell infiltration and demyelination in the optic nerve strongly correlated with the severity of clinical disease. This was accompanied by retinal ganglion cell loss and glia and macrophage activation as well as IL-6 secretion in EAE animals.

## Methods

### Animals

Male C57BL/6 mice (8 weeks old; ZVTE, Mainz, Germany) were housed and maintained under environmentally controlled conditions in a 12-hour light/dark cycle with access to food and water *ad libitum* in the absence of pathogens. All experiments that involved animal use were performed in compliance with the ARVO statement for the Use of Animals in Ophthalmic and Vision Research. These experiments have been approved by the local animal care committee.

### Induction and evaluation of experimental autoimmune encephalomyelitis

Mice (*n* = 9) were injected subcutaneously in the tailpleat with 100 μg MOG_35-55_ (MOG) in complete Freund’s adjuvant (CFA, 550 μg/100 μl, Difco Laboratories, Detroit, MI, USA). At the same time as immunization and 48 hours post immunization, animals received 200 ng of pertussis toxin intraperitoneally (Sigma-Aldrich, St Louis, MO, USA). Control mice (CO; *n* = 8) received the same treatment as immunized mice except that MOG was replaced by PBS.

All animals were examined and scored daily for clinical signs of EAE [17]. Animals were clinically graded as follows: 0 = no signs, 1 = loss of tail tonicity, 2 = loss of tail tonicity and mild paralysis of hindlimbs, 3 = paralysis of hindlimbs, 4 = hindlimb paralysis and mild paralysis of forelimbs and 5 = complete paralysis or death.

### Cresyl violet staining of flatmounts

**Figure 1:**
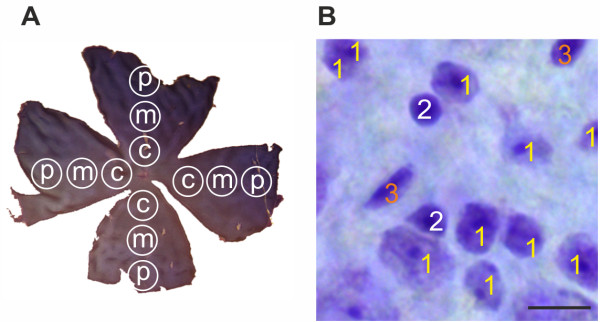
**Areas and cell types of interest for data analysis. (A) **Representative flatmount preparation showing three areas: central (c), middle (m) and peripheral (p). Photographs of each area in each arm were taken using an Axio Imager M1 microscope. **(B) **The pictures were analyzed using ImageJ software (1: neurons, 2: glia, 3: endothelia). Only neurons were included for further analysis. Scale bar: 10 μm.

Then 23 days post immunization, eyes (*n* = 8 per group) were fixed in 4% paraformaldehyde for 1 h and then prepared as flatmounts. After de- and rehydration in 70% to 100% ethanol, retinal flatmounts were stained with Nissl stain with 1% cresyl violet (Merck, Darmstadt, Germany) [6]. Subsequently, all slides were again dehydrated in ethanol followed by incubation in xylene, before flatmounts were mounted with Eukitt (both Merck, Darmstadt, Germany). Photographs of the central, middle and peripheral parts of each flatmount arm were taken with a microscope equipped with a CCD camera (Axio Imager M1, Zeiss, Oberkochen, Germany) using 400× magnification (Figure 1A). The Nissl staining allowed distinction between neuronal cells (>8 μm in diameter, irregular cell shape and prominent nucleolus), endothelial cells (longitudinal shape) and glia cells (<8 μm, round shape) based on their morphology, size and location (Figure 1B) [6]. Neuronal cells were counted in a blinded fashion in the three areas of each arm using ImageJ (Version 1.44, NIH, Bethesda, MD, USA); the other cell types were excluded from the further analysis.

### Histology and morphology of retina crosssections

Hematoxylin and eosin (H&E) staining was performed to analyze the thickness of the retinal layers. Additionally, Bielschowsky’s silver impregnation (BSI) was used to detect deposits and infiltrates in the retina. The eyes of control and EAE animals were fixed in 4% paraformaldehyde and embedded in paraffin (*n* = 5 to 7 eyes/group). Retina crosssections (5 μm thick) were cut. After de- and rehydration in 70% to 100% ethanol, the crosssections were stained with H&E and BSI [18,19]. Subsequently, all slides were again dehydrated in ethanol followed by incubation in xylene before being mounted with Eukitt.

Measurement of retinal layers was conducted as follows. Pictures of six H&E stained crosssections per eye were taken with a microscope equipped with a CCD camera (Axio Imager M1) at 40× magnification. Images were analyzed with the built-in measuring tool in ZEN 2011 software (V1.0.1.0, Zeiss, Oberkochen, Germany). The thicknesses of the whole retina (excluding the outer segments) and the ganglion cell layer (GCL), inner plexiform layer (IPL), inner nuclear layer (INL), outer plexiform layer (OPL) and outer nuclear layer (ONL) were measured for each layer in each picture in three different areas. Data were collected in Excel and then imported to Statistica (V10.0; Statsoft, Tulsa, OK, USA) for statistical analysis.

### Immunohistochemistry of retina crosssections

Paraffin-embedded retina crosssections (5 μm thick, *n* = 5 to 7 eyes/group) were prepared for immunohistochemistry as follows: sections were deparaffinized and rehydrated. Antigen retrieval was performed in 10 mM sodiumcitrate buffer (pH 6.0) containing 0.05% Triton X-100 at 95°C in a water bath for 30 min. The sections were then blocked in 10% appropriate serum in 0.1% Triton X-100 in PBS. Six retina crosssections per animals were used for each staining. Retinal ganglion cells were stained with Brn-3a (1:100, Santa Cruz, Heidelberg, Germany), a specific retinal ganglion cell marker. Inactive caspase 3 (1:100, Biozol, Echingen, Germany), an apoptosis protein marker, was used to stain apoptotic cells. Both were labeled with the secondary antibody Alexa 555 (1:500, Invitrogen, Darmstadt, Germany). Macroglial cells were investigated with an Alexa 488 conjugated glial fibrillary acidic protein (GFAP) antibody (1:1200, Millipore, Billerica, MA, USA). Microglial cells were stained with Iba1 (1:400, Wako Chemicals, Neuss, Germany) followed by Alexa 555 (1:500, Invitrogen) as the secondary antibody. Staining of activated microglial cells was performed in a double staining experiment using F 4/80 antibody (1:50, AbDserotec, Düsseldorf, Germany) and Iba1 (1:400, Wako Chemicals). Appropriate secondary antibodies were used (Alexa 488, 1:500, Life Technologies Darmstadt, Germany; Alexa 555, 1:500, Invitrogen). All slides were mounted with antifade medium containing fluorescent nuclear stain DAPI (Fluoro-Mount w/DAPI; Dianova). In general, two photographs were taken from the periphery, middle and central part of each crosssection with an AxiocamHRc CCD camera on a Zeiss Imager M1 fluorescence microscope using a 40× objective. The digitalized images were transferred to Corel PaintShop Photo Pro (V 13; Corel Corporation, Fremont, CA, USA) and excerpts (800 × 610 pixels) including the GCL and INL were cut out.

Using the photographs of the Brn3a and caspase 3 stained sections, RGCs or caspase 3^+^ cells were counted in six photographs of six retina crosssections per animal, as described above, in a masked fashion in the GCL using ImageJ. The data was collected in Excel and then imported into Statistica for further analysis.

For the glial fibrillary acidic protein (GFAP) staining, the same camera settings, including exposure time, were applied on all images using a Zeiss Imager M1 fluorescence microscope equipped with an AxiocamHRc CCD camera. As described above, six photographs of six retina crosssections per eye were made and the GFAP channel was transferred into ImageJ for further analysis [20]. To automate the data analysis, we used a macro that we wrote ourselves. First, the image was transformed into greyscale. After background subtraction (20 pixels) the upper and lower thresholds were set (lower threshold: 3.5; upper threshold: 80). Background subtraction and upper and lower thresholds represent mean values of the manually analyzed images of both CO and EAE animals. The percentage of the labeled GFAP^+^ area and the staining intensity were measured for each picture using a ImageJ macro, imported to Excel and transferred to Statistica for further analysis.

Secretion of interleukin-6 was investigated with an IL-6 antibody (1:100, Abcam, Cambridge, UK) using FITC as the secondary antibody (1:500, Abcam). Sections were co-labeled with GFAP (1:400, Millipore) and Cy3 as the secondary antibody (1:500, Millipore). We counted the IL-6 positive vesicles in the GCL of the whole retina crosssections (*n* = 5 to 6 sections/eye) with an AxiocamHRc CCD camera on a Zeiss Imager M1 microscope equipped with a 40× objective. The data were collected in Excel and then imported into Statistica for analysis.

### Optic nerve histology

Optic nerves were fixed in 4% paraformaldehyde overnight and then embedded in paraffin. Longitudinal optic nerve sections, 5 μm thick, were obtained. To investigate alterations and structural changes, the optic nerve sections were stained with standard H&E (*n* = 4 per group) and luxol fast blue (LFB; *n* = 5 per group) [3,6]. Subsequently, all slides were dehydrated and embedded using the same dehydration and embedding protocol as described above for retina crosssections.

Inflammatory cell infiltration in longitudinal sections of the optic nerves stained with H&E was evaluated [1-3,13,21]. Three areas of each optic nerve (four sections/animal) were graded according to a scale from 0 to 4 by a masked observer: 0 = no infiltration, 1 = mild cellular infiltration of the optic nerve or optic nerve sheath, 2 = moderate infiltration, 3 = severe infiltration and 4 = massive infiltration of the optic nerve parenchyma and nodule infiltration. The average score for each optic nerve was used for statistical analysis.

The demyelination grade was evaluated on longitudinal LFB-stained optic nerve sections [21-23]. Four slices per animal were used for LFB staining. Three areas of each optic nerve (four sections/animal) were graded as follows: 0 = no demyelination, 1 = moderate demyelination and 2 = severe demyelination. The average score for each optic nerve was used for later statistical evaluation.

### Statistics

Data are presented as mean ± standard error of the mean (SEM) unless otherwise noted. Histology data for the two groups were compared using the two-tailed Student’s *t*-test (Statistica). *P *values below 0.05 were considered statistically significant.

## Results

### Immunization with MOG leads to clinical experimental autoimmune encephalomyelitis

**Figure 2:**
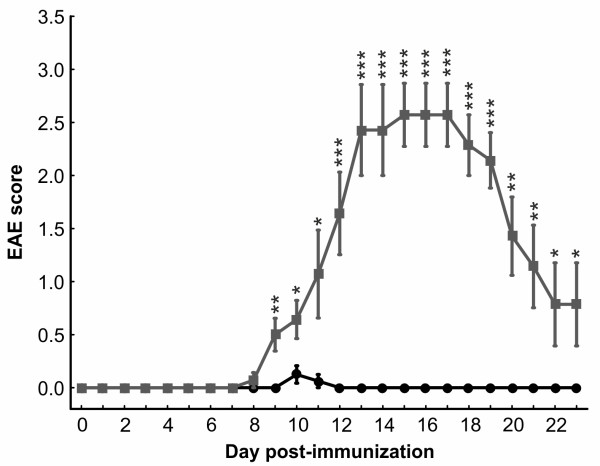
**Scores used for evaluation of EAE symptoms.** Mice were examined daily using a clinical EAE scoring system ranging from 0 (no signs) to 5 (complete paralysis). The clinical symptoms were scored for 23 days. Symptoms peaked at day 15 (CO: 0 ± 0, EAE: 2.6 ± 0.3, *P*< 0.0001) and started to decline at day 17 post immunization. The abscissa represents the experimental days and the ordinate represents the severity of clinical symptoms (*n* = 8 to 9 animals/group). Black=CO, gray=EAE; *: p<0.05; **: p<0.01; ***: p<0.001. CO, control; EAE, experimental autoimmune encephalomyelitis.

Animals first developed symptoms of clinical EAE, indicated by a loss of tail tonicity, 8 days post immunization (Figure 2). Between days 15 and 17 (both days: CO: 0 ± 0, EAE: 2.6 ± 0.3, *P*< 0.0001) the animals displayed the strongest clinical EAE symptoms with loss of tail tonicity and paralysis of the hindlimbs (Figure 2). After day 17 the clinical symptoms of EAE animals started to decline. But they were significantly increased compared to CO animals at all points in time starting at 8 days post immunization (Figure 2).

### Inflammation and demyelination in the optic nerve

**Figure 3:**
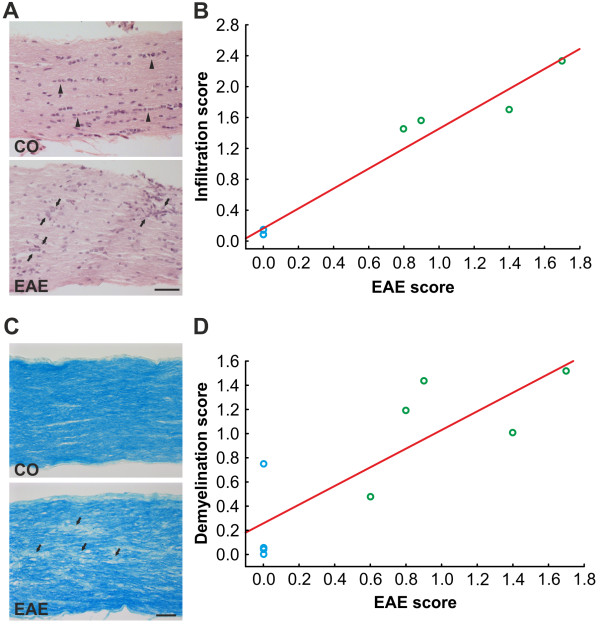
**Evaluation of changes in the optic nerve. (A) **H&E-stained longitudinal optic nerve sections of EAE animals exhibited signs of optic neuritis indicated by cell clustering and disorganization (arrowheads) compared to a regular alignment of cells in the CO (arrows). **(B) **The infiltration score (from 0 for no infiltration to 4 for massive infiltration) positively correlated with the EAE score (*P*< 0.0001, *r* = 0.9824). CO mice (blue circles) had no clinical symptoms and infiltration while EAE mice (green circles) had both clinical symptoms and infiltration of cell clustering in the optic nerve (*n* = 4 eyes/group). **(C) **Luxol fast blue staining in longitudinal optic nerve sections of EAE animals revealed signs of demyelination indicated by white areas in the optic nerve (arrowheads). In CO nerves the myelin sheaths were uniformly stained. **(D) **The demyelination score (from 0 for no demyelination to 2 for strong demyelination) was positively correlated with the EAE score (*P* = 0.0053, *r* = 0.8328). CO animals displayed no signs of clinical EAE while in contrast all EAE mice had a clinical EAE score as well as mild to severe demyelination in the optic nerve (*n* = 5 eyes/group). Scale bars: 50 μm. CO, control; EAE, experimental autoimmune encephalomyelitis; H&E, hematoxylin and eosin stain.

To assess structural and morphological changes of the optic nerves, H&E (Figure 3A) and LFB staining (Figure 3C) were performed 23 days post immunization. While the cell nuclei of the axonal fibers were regularly lined up in the optic nerve (ON) of CO mice, they were clustered in the nerves of EAE animals (Figure 3A). Furthermore, massive infiltrate deposits were observed throughout the ON of animals that developed EAE (Figure 3A). The severity of the infiltrates were significantly higher in EAE (*P* = 0.0047) and positively correlated with the clinical EAE score (*r* = 0.98, *P*< 0.0001, *r*^2^ = 0.97, *y* = 1.2905*x* + 0.1642, Figure 3B).

The structure of the ON of CO mice stained with LFB displayed a uniform staining of the myelin sheaths and a smooth distribution of the optic nerve fibers (Figure 3C). The LFB staining of the ON of EAE mice showed some whitening, indicating a loss of the myelin sheaths (Figure 3C). The axonal fibers of EAE mice exhibited a more disorganized, wavy structure (Figure 3C). The severity of the demyelination was significantly higher in EAE (*P* = 0.0018) and was positively correlated with the clinical EAE score (*r* = 0.83, *P* = 0.0053, *r*^2^ = 0.69, *y* = 0.7719*x* + 0.258, Figure 3D). Our results indicate that optic nerve damage is strongly correlated with the severity of clinical EAE.

### Ganglion cell loss in experimental autoimmune encephalomyelitis animals

**Figure 4:**
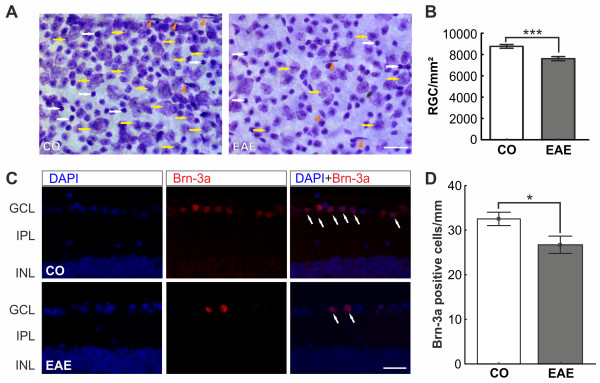
**RGC count in retina flatmounts and retina crosssections. (A) **Representative Nissl-stained flatmounts of CO and EAE retinas reveal a loss of neurons (yellow arrows) in EAE retinas. The white arrows show glia cells and the arrowheads endothelial cells. Scale bar: 20 μm.**(B) **Quantification of neurons on retinal flatmounts (*n* = 8 eyes/group). The number of neurons (*P*< 0.0001) was significantly reduced in the EAE group after 23 days. **(C) **Immunohistochemical staining of CO and EAE retinas with Brn-3a (RGCs, red) and DAPI (nuclei, blue) 23 days post immunization. The merged picture shows a reduction of Brn-3a^+^ stained RGCs (arrows) in the GCL of EAE animals. **(D) **Quantification of Brn-3a^+^ cells in the retinas of CO and EAE animals (*n* = 5 to 7 eyes/group). RGC numbers were significantly reduced in the retinas of EAE mice (CO: 32.5 ± 1.5 cells/mm, EAE: 26.7 ± 1.9 cells/mm, *P* = 0.0170). Scale bar: 25 μm. *: p<0.05; ***: p<0.001. CO, control; EAE, experimental autoimmune encephalomyelitis; GCL, ganglion cell layer; INL, inner nuclear layer; IPL, inner plexiform layer; RGC, retinal ganglion cell.

Neuronal cells of CO and EAE animals, defined as light purple cells with a dark nucleus and a rough border, were quantified on Nissl-stained flatmounts (Figure 4A). We observed a significant loss of neuronal cells in EAE mice (7,611.9 ± 194.1 cells/mm^2^) compared to CO (8,763.1 ± 177.5 cells/mm^2^, *P*< 0.0001; Figure 4B). Nissl unspecifically labels all neuronal cell types, like RGC and amacrine cells, in the GCL of the retina. Therefore, an additional staining was performed. Retina crosssections were labeled with the specific RGC marker Brn-3a (Figure 4C). Quantification of Brn-3a^+^ cells revealed a significant reduction of RGCs in EAE animals (CO: 32.5 ± 1.5 cells/mm,EAE: 26.7 ± 1.9 cells/mm, *P* = 0.0170, Figure 4D). Consequently, EAE immunization causes to a loss of neuronal cells in the retina, especially RGCs.

### Intact retinal structures after immunization

**Figure 5:**
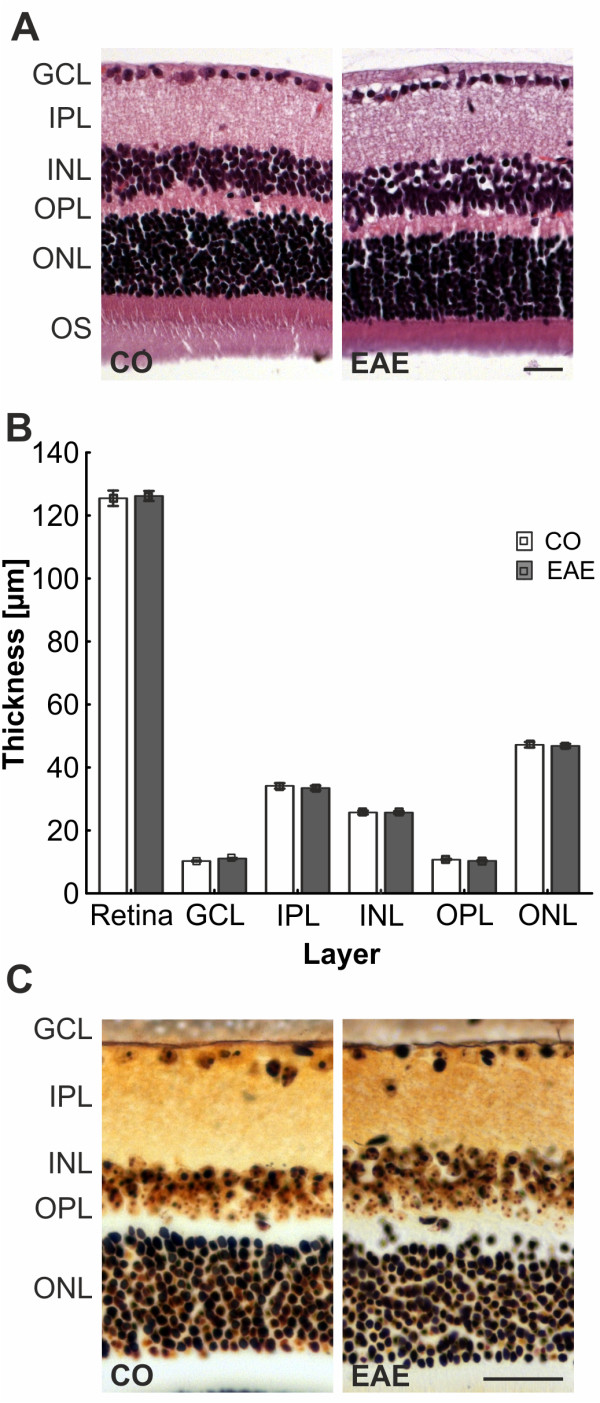
**Evaluation of infiltrates and neuronal plaques in the retina. (A) **H&E-stained retina crosssections of CO (left) and EAE animals (right, *n* = 5 to 7 eyes/group). No difference between CO and EAE in retinal structure and no neuronal tangles and plaques were observed. **(B) **Quantification of the retinal layer thickness (*n* = 5 to 7 eyes/group). No difference was noted in the thickness of the layers. **(C) **Bielschowsky’s silver stain of retina sections of CO and EAE animals (*n* = 5 to 7 eyes/group). The retinal structure of EAE animals was not altered compared to CO and no neuronal plaques were noted. Scale bars: 25 μm. CO, control; EAE, experimental autoimmune encephalomyelitis; GCL, ganglion cell layer; INL, inner nuclear layer; IPL, inner plexiform layer; ONL, outer nuclear layer; OPL, outer plexiform layer; OS, outer segments.

To investigate if the structure and morphology of the retina stayed intact after EAE immunization we performed H&E and silver staining on retina crosssections. We did not observe structural or morphological changes in H&E-stained section of EAE or CO animals (Figure 5A). Quantification of the thickness of the whole retina revealed no difference between the control (125.5 ± 2.5 μm) and EAE group (126.2 ± 1.6 μm; *P* = 0.8). Also, despite the RGC loss (Figure 4B), no decrease in the thickness of the GCL was noted (CO: 10.3 ± 0.3 μm, EAE: 11.1 ± 0.3 μm, *P* = 0.09, Figure 5B). Only apoptosis of cells occurs in the GCL and there is no destruction of this layer. Furthermore, no changes in thickness were noted for the plexiform layers: IPL (CO: 34.1 ± 0.9 μm, EAE: 33.5 ± 0.7 μm, *P* = 0.5) and OPL (CO: 10.7 ± 0.3 μm, EAE: 10.3 ± 2.2 μm, *P* = 0.25) (Figure 5B). The nuclear layers, INL (CO: 25.7 ± 0.6 μm, EAE: 25.7± 0.4 μm, *P* = 0.98) and ONL (CO: 47.2 ± 0.9 μm, EAE: 46.8 ± 0.7 μm, *P* = 0.74) also display no alteration in thickness in EAE animals compared to controls (Figure 5B). Although immunizing with MOG_35-55_ led to RGC loss it did not affect the structure and morphology of the retina 23 days after immunization, which would be a sign of retinal remodeling and degeneration.

### Increased apoptosis in experimental autoimmune encephalomyelitismice

**Figure 6:**
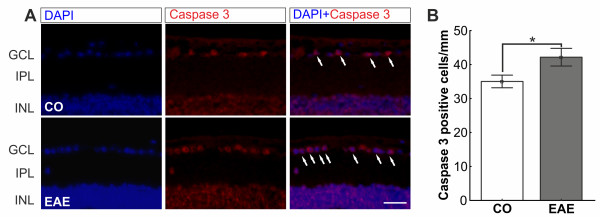
**Apoptosis in retina crosssections. (A) **Immunohistochemical staining of CO and EAE retinas with caspase 3^+^ (apoptosis protein, red) and DAPI (nuclei, blue) 23 days post immunization. In the merged image more caspase 3^+^ cells (arrows) are visible in the GCL of EAE animals. **(B) **Mean cell count of caspase 3^+^ cells in the GCL of the retinas of CO and EAE animals at 23 days (*n* = 5 to 7 eyes/group). More caspase 3^+^ cells were noted in the retinas of EAE animals (CO: 35.0 ± 1.9 cells/mm, EAE: 42.2 ± 2.6 cells/mm, *P* = 0.0222). Scale bar: 25 μm. *: p<0.05. CO, control; EAE, experimental autoimmune encephalomyelitis; GCL, ganglion cell layer; INL, inner nuclear layer; IPL, inner plexiform layer.

In the GCL of both groups, caspase 3^+^ cells were present (Figure 6A). Quantification of caspase 3^+^ cells in the retinal ganglion cell layer of CO (35.0 ± 1.9 cells/mm) and EAE (42.2 ± 2.6 cells/mm) mice demonstrated significantly more caspase 3^+^ cells in EAE animals at 23 days post immunization (*P* = 0.0222, Figure 6B). This implies that more apoptotic RGCs are present in EAE mice.

### Gliosis in experimental autoimmune encephalomyelitis retina

**Figure 7:**
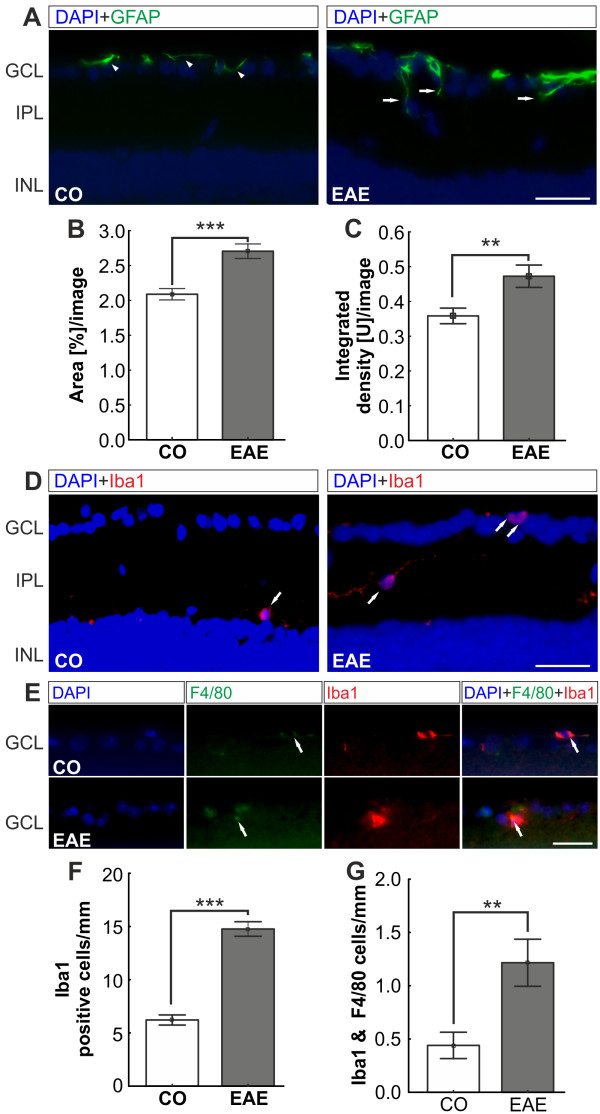
**Macroglial and microglial changes in EAE retinas. (A) **Representative images of GFAP-stained CO and EAE retinas. The GFAP^+^ signal was only present in the Müller cell end-feet of CO animals (arrowheads) while it stretched into the IPL of EAE animals (arrows). **(B) **Quantification of the GFAP^+^ area (*n* = 5 to 7 eyes/group). EAE animals expressed significantly more GFAP^+^ protein compared to CO animals (*P*< 0.0001). **(C)**Quantification of fluorescence signal. EAE animals had significantly more GFAP^+^ signal than CO animals (*P* = 0.0033). **(D) **Iba1 staining of CO and EAE retinas. Iba1^+^ cells were predominantly located in the IPL of CO and EAE animals, but were more abundant in EAE animals. **(E) **Iba1 and F4/80 double staining of retina sections. Only a few microglial cells (Iba1, red) were also activated as macrophages in both groups (arrows). **(F) **Mean number of Iba1^+^ cells of retina crosssections (*n* = 5 to 7 eyes/group). More Iba1^+^ cells were located in the retinas of EAE animals (CO: 6.2 ± 0.5 cells/mm, EAE: 14.8 ± 0.7 cells/mm, *P*< 0.0001). **(G) **Statistical analysis showed significantly more Iba1 and F 4/80 co-labeled cells in EAE retinas (CO: 0.44 ± 0.12 cells/mm, EAE: 1.22 ± 0.22 cells/mm, *P* = 0.0023).Scale bars: 25 μm. **: p<0.01; ***: p<0.001. CO, control; EAE, experimental autoimmune encephalomyelitis; GCL, ganglion cell layer; GFAP, glial fibrillary acidic protein; INL, inner nuclear layer; IPL, inner plexiform layer.

To investigate the extent of gliosis in the retina of control and immunized mice, we examined the expression of GFAP in astrocytes and end-feet of Müller cells on retina crosssections. These cells become reactive glial cells when pathological changes like inflammation occur [24,25]. GFAP expression was analyzed for control and immunized mice to determine whether changes can be noted. Subsequently, 23 days after immunization only discrete GFAP staining was observed in Müller end-feet and astrocytes in the nerve fiber layer and GCL of control animals, while in EAE animals the expression of GFAP reached into the IPL (Figure 7A). Quantification of the GFAP^+^ area revealed a significant GFAP increase in EAE animals (CO: 2.1 ± 0.1, EAE: 2.7 ± 0.1, *P*< 0.0001, Figure 7B) as well as a significant increase in the strength of the fluorescent signal (CO: 0.4 ± 0.02, EAE: 0.5 ± 0.03, *P*< 0.0033, Figure 7C). This indicates an increased expression of fibrillary acidic protein accompanied with an activation of astrocytes triggered by inflammation and apoptosis of RGCs.

### Activation of microglial cells in experimental autoimmune encephalomyelitis mice

We investigated the microglial responses in the retina of immunized and control animals as a sign of retinal inflammation. The microglial cells were analyzed using a specific antibody against the calcium-binding protein Iba1, a protein highly expressed in microglial cells [26,27], on retina crosssections. Significantly more Iba1^+^microglial cells were counted in the retinas of EAE animals 23 days after immunization (CO: 6.2 ± 0.5 cells/mm, EAE: 14.8 ± 0.7 cells/mm, *P*< 0.0001, Figure 7D, F). This could be a response to an earlier retinal inflammation.

To investigate the functional state of the microglial cells, a co-labeling of microglial cells with Iba1 and the receptor binding antibody F 4/80 was performed. Only a few Iba1 and F 4/80 positive cells were noted in both groups (CO: 0.44 ± 0.12 cells/mm, EAE: 1.22 ± 0.22 cells/mm, Figure 7E). But significantly more Iba1 and F 4/80^+^ cells were present in EAE retinas (*P* = 0.0023, Figure 7G). Consequently, more macrophages were activated in the retinas of EAE animals compared to CO.

### Increased interleukin-6 expression in experimental autoimmune encephalomyelitis animals

**Figure 8:**
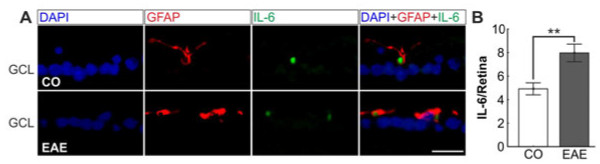
**Interleukin-6 secretion in the retina. (A) **Sections of CO and EAE mice were double stained with IL-6 (green) and GFAP (red). The merged image shows IL-6 positive vesicles encapsulated by macroglial (GFAP) processes in CO and EAE animals. Scale bar: 20 μm. **(B) **Significantly more IL-6 positive vesicles were noted in EAE retinas (8.0 ± 0.8 vesicles/retina) compared to CO (4.9 ± 0.5 vesicles/retina, *P* = 0.0072). **: p<0.01; CO, control; EAE, experimental autoimmune encephalomyelitis; GCL, ganglion cell layer; GFAP, glial fibrillary acidic protein; IL-6, interleukin-6.

To examine the role of cytokines, we investigated the presence of IL-6. Double staining with GFAP revealed that IL-6 positive vesicles were encapsulated by macroglial processes (Figure 8A). Significantly more IL-6 positive vesicles were detected in EAE retinas (8.0 ± 0.8 vesicles/retina) then CO retinas (4.9 ± 0.5 vesicles/retina, *P* = 0.0072, Figure 8B). The increased activation of macroglial cells and the higher production of IL-6 in EAE retinas point to the conclusion that inflammatory processes in the eye play a role during EAE.

## Discussion

In this study we investigated the effects of EAE in the optic nerve as well as in the retina. We noted that the grade of clinical EAE determined the strength of optic neuritis. This neuritis led to an RGC loss in the retina. As a consequence of this loss, inflammatory processes were activated.

### Optic neuritis in experimental autoimmune encephalomyelitis animals

Immunization with MOG_35-55_ leads to typical signs of optic nerve inflammation like cell clustering, disruption of the axon fiber organization and demyelination [1-3,6,13]. We discovered that demyelination and infiltration of the optic nerve of EAE mice strongly correlated with the severity of the clinical disease score (Figure 3). To our knowledge this is the first time that this relation between clinical diagnosis and cellular response in the optic nerve has been reported for this MOG EAE model. Interestingly, we also noted a remission of the clinical symptoms 17 days post immunization. This indicates cessation of inflammation activity and a possible restoration of the myelin sheaths. Indeed, in models of multiple sclerosis a complex process becomes activated. Macroglial and microglial cells are recruited during the demyelination processes to remove damaged myelin. This myelin clearing is crucial in stopping the inflammation and starting the remyelination process in the central nervous system, especially in the early disease stages [28-30]. But this process becomes less effective in later disease stages and leads to scar-like processes like building of plaques around the axons [31]. Additionally, T cells are activated in multiple sclerosis and are known to secrete cytokines such as TNF-α and IFN-γ [31,32]. T cells and microglial cells are activated and play a major role in the demyelination of the optic nerve [11]. We assume that the demyelination and scarring process noted in our study as well as the secretion of cytokines leads to severe damage of the RGC axons.

### Apoptosis of retinal ganglion cells

Axonal damage often results in axonal degeneration and permanent loss of the cell body by apoptosis. In optic nerve crush or optic nerve transection models, damaging the optic nerve leads to a rapidly induced stress response in RGCs that finally results in apoptosis of the neurons [33-35]. In our study, immunization with MOG_35-55_ led to significant RGC loss (*P*< 0.0001, Figure 4) through apoptosis (*P* = 0.0222, Figure 6). The MOG EAE model probably has a later onset of apoptosis, compared to other EAE mouse models [2,3]. This may either indicate that different antigens induce a different strength or phenotype of inflammation responses or that different mouse strains have different levels of resistance to RGC loss based on their genetic background. It has been demonstrated that the mouse strain significantly influences the starting point of RGC loss in an optic nerve crush model [36]. This could also affect the onset of EAE.

### Changes in retinal layers

No difference in the thicknesses of the retina layers were observed between control and EAE animals (Figure 5). In recent years, optical coherence tomography (OCT) has become a common method for non-invasively observing and investigating retinal morphology in rodents [37-39]. Although the major layers of the retina can be identified using OCT, there are challenges in applying this method in rodents. It is rather difficult to visualize and monitor changes in the nerve fiber layer of a retina because of its thin and fine structure, especially since all commercially available systems today were designed for human eyes. Therefore, we did not use OCT to analyze the retinal structure. Instead, we performed H&E staining to quantify the thickness of the retinal layers (Figure 5B).

### Glia cell and cytokine response

In a previous study, the number of RGCs in eyes with severe inflammation was significantly lower than the number of RGCs in eyes with mild inflammation [40]. This confirms that optic neuritis is not merely an inflammatory condition, but also involves significant neurodegeneration mechanisms. Our results indicate that, as a consequence of optic nerve axon damage and apoptosis of RGCs, the macroglia becomes activated (Figure 7A,B,C). For other retinal diseases, like age-related macular degeneration, diabetic retinopathy or glaucoma, macroglial activation after neuronal apoptosis, especially of RGCs, has been described [41-43]. The common pathway in those diseases seems to be the expression of neurotoxic molecules like interleukins by astrocytes and Müller glia, which lead to an inflammatory RGC loss [41-44]. The occurrence of inflammatory processes in the retina caused by EAE is supported by the presence of higher levels of IL-6 closely associated with GFAP in EAE animals in our study (Figure 8). We assume that these mechanisms are also triggered in the EAE animal model.

Additionally, due to an inflammatory event or cellular stress, microglial cells are rapidly activated and start to migrate to the location of the event. This has been described for neuronal injuries, ischemia, metabolic as well as hereditary retinopathies and photoreceptor degeneration [45-48]. After neuronal injury, the microglia is activated and releases pro inflammatory substances like TNF-α and interleukins. We noted significantly more microglial cells in EAE retinas (*P*< 0.0001, Figure 7D, E). We assume that the activation of the microglia (Figure 7E and G) is a direct response to RGC degeneration. We cannot rule out that other mechanisms lead to an increase of glial responses in the retina. The disruption of the blood–brain barrier is a fundamental event in the pathogenesis of symptoms of primary central nervous demyelination such as EAE [49,50]. Furthermore, this disruption leads to inflammatory processes in an experimental autoimmune uveitis model [51,52]. This might also be the case in EAE. In age-related macular degeneration, macrophages are present during the early stages of the disease and can be directly associated with RGC loss due to phagocytosis of the cell deposits [53]. In our study, we detected few macrophages in EAE and control animals. However, we noted significantly more macrophages in EAE mice. Future studies are needed to investigate if higher macrophage activation is observed at earlier points in time.

## Conclusion

Immunization with MOG_35-55_ caused clinical EAE symptoms in mice. For the first time, we described a strong correlation between the severity of optic nerve damage with the grade of clinical EAE symptoms. Also, the structure of the retina was still intact, we noted a significant RGC loss and an activation of macro- and microglial cells in the retina. At this late point in time, few microglial cells were activated as macrophages, probably due to the apoptosis of the RGCs. The inflammation was presumably triggered by macroglial secretion of interleukin-6. Based on our findings, optic neuritis in EAE can be described as a disease of three continuous mechanisms. First, EAE starts as an autoimmune disease by causing neuroinflammatory responses against the myelin sheaths. This results in demyelination and inflammation in the optic nerve. In the second phase, it continues as a neurodegenerative disease noticeable by apoptosis of RGCs due to demyelination of the axons. These two steps have already been suggested and discussed by others [3]. We hypothesize that this leads to the third step. The apoptosis of RGCs induces to an activation of inflammatory processes in the retina. These include the production of IL-6, the activation of astrocytes and microglial cells and gliosis.

## Abbreviations

BSI: Bielschowsky’s silver impregnation; CFA: Complete Freund’s adjuvant; CO: Control; EAE: Experimental autoimmune encephalomyelitis; GCL: Ganglion cell layer; GFAP: Glial fibrillary acidic protein; H&E: Hematoxylin and eosin stain; IFN: Interferon; IL-6: Interleukin-6; INL: Inner nuclear layer; IPL: Inner plexiform layer; LFB: Luxol fast blue; MOG: MOG_35-55_peptide; MS: Multiple sclerosis; OCT: Optical coherence tomography; ON: Optic nerve; ONL: Outer nuclear layer; OPL: Outer plexiform layer; OS: Outer segments; PBS: Phosphate-buffered saline; RGC: Retinal ganglion cell; SEM: Standard error of the mean; TNF: Tumor necrosis factor.

## Competing interests

The authors declare that they have no competing financial or personal interests, and that none of the authors’ institutions have contracts relating to this research through which they may stand to gain financially now or in the future.

## Authors’ contributions

LH and HS established the protocols, carried out experiments, performed the data analysis and statistical analysis, and drafted the manuscript. FCK and APH established protocols, carried out experiments and revised the manuscript. HBD drafted and revised the manuscript. SCJ designed the study, carried out experiments and drafted the manuscript. All authors read and approved the final manuscript.
